# An international survey of recalcitrant and recurrent tinea of the glabrous skin—A potential indicator of antifungal resistance

**DOI:** 10.1111/jdv.20146

**Published:** 2024-07-13

**Authors:** Sidra S. Khan, Roderick Hay, Ditte Marie L. Saunte

**Affiliations:** ^1^ The European Academy of Dermatologists and Venerologist's Mycology Taskforce Lugano Switzerland; ^2^ The Dermatology Department, Withington Community Hospital Manchester University Foundation Trust Manchester UK; ^3^ The Department of Clinical Sciences and International Public Health The Liverpool School of Tropical Medicine Liverpool UK; ^4^ King's College London London UK; ^5^ Department of Dermatology Zealand University Hospital Roskilde Denmark; ^6^ Department of Clinical Medicine, Faculty of Health and Medical Sciences University of Copenhagen Copenhagen Denmark

## Abstract

**Background:**

There has been a global rise in cases of dermatophytosis and, in particular, recalcitrant and recurrent cases on tinea of the glabrous skin. This phenomenon, particularly prevalent in India, has been linked to the concerning rise of antifungal resistance. The challenge is amplified by a dearth of comprehensive, international data to understand the global scope and characteristics of such cases.

**Objectives:**

This study aims to collate international insights, focusing on areas outside Europe (as this was previously published), to map the extent and characteristics of clinical and, where possible, laboratory confirmed tinea of the glabrous skin through an online survey administered to dermatologists globally.

**Methods:**

An online survey was distributed from February 2022 to July 2023 and captured data on respondents' experience of recalcitrant and recurrent tinea of the glabrous skin over the preceding 3 years.

**Results:**

A total of 260 responses were received spreads across 36 countries, excluding Europe. In total, 91.7% reported seeing cases of recalcitrant or recurrent tinea of the glabrous skin over the preceding 3 years. Common anatomical sites affected were the trunk and groin. *T. mentagrophytes* and *T. rubrum* were the predominant species implicated, and there were low rates of laboratory confirmed dermatophyte resistance.

**Conclusions:**

The high rates of reported recalcitrant and recurrent tinea of the glabrous skin underscore an urgent need for global collaborative efforts and enhanced diagnostic measures. The findings advocate for the establishment of a standardized global disease registry, regulation of over‐the‐counter antifungal and steroid combinations, correlation of clinical suspicion with laboratory confirmed drug resistance and exploration of alternative therapeutic strategies to mitigate the burgeoning challenge of dermatophyte resistance.

## INTRODUCTION

Dermatomycoses, defined as fungal infections of the skin, hair and nails, are very common, affecting almost 1 billion people worldwide.^1^ The burden is highest amongst countries with a low sociodemographic index (SDI) and some of the most vulnerable in society, such as children aged 1–5 and the elderly.^2^

Amongst these infections, dermatophytes, also known as tinea or ‘ring worm’, are a prevalent group of superficial filamentous fungi with a predilection for keratin rich tissue. Often considered ‘mild’ in nature, they can lead to a significant impact on quality of life.^3^ They are typically amenable to topical or oral antifungal therapy but there has been a rising number of cases of dermatophyte resistance to antifungals, which manifests as recalcitrant or recurrent infections, causing great concern amongst the dermatology community globally.^4^

Treatment failure can be attributed to a number of factors including poor concordance due to cost of medications and misuse of over‐the‐counter combinations of topical antifungals and potent steroids, increasing rates of host immune dysfunction and variability in the quality of antifungal drugs in some regions.^5^, ^6^ Arguably, the most concerning reason of all is the rising number of cases of clinically suspected dermatophyte resistance, which manifests as recalcitrant or recurrent infections, and dermatophyte infections with proven antifungal resistance globally.^7^

India has seen a significant increase in dermatophytosis cases and treatment failures over the past decade.^6^, ^7^, ^8^ True incidence and prevalence rates are difficult to determine as robust prevalence studies are lacking. Nonetheless, data from reports of the prevalence of dermatophytosis range from 6.1% in community based surveys to 61.5% in hospital‐based studies across parts of the country.^6^ One study from a tertiary centre in the North West India of cases of clinically diagnosed dermatophytosis noted that the percentage of patients with chronic (>3 months duration) disease to be as high as 62.5%.^9^ Most of these are caused by the dermatophyte predominantly associated with the Indian outbreak, *Trichophyton (T.) indotineae*, which can only be identified using molecular tools. This strain is often associated with high rates of terbinafine resistance due to mutations in the squalene epoxidase gene.^4^

Cases of recalcitrant or recurrent dermatophytosis in India have been reported in all age groups. Initially, it was noted to occur more frequently in men, but recent studies suggest a trend moving towards increasing frequency in women.^10^, ^11^, ^12^ It has also been described occurring in familial clusters, and this is something our authors have noted anecdotally as well, from other countries in the region.

Clinically, Indian cases have been described as widespread with varying degrees of inflammation. The morphology has also been noted to be far more striking in some cases with multiple annular lesions, large plaques and a ‘pseudoimbricata’ type pattern being observed within the literature.^6^ The clinical disease progression has been noted to be abrupt by some authors, and interestingly, a ‘rebound’ flare and even pustulation has been described in some cases upon starting oral itraconazole.^6^

Cases are now being reported outside of India, in other parts of Asia, northern Africa, Australia, North America and Europe.^13^ *Saunte* et al confirmed the presence of clinical and/or mycological confirmed antifungal resistance across most of Europe and two recent cases garnered national attention in the United States.^14^, ^15^ What was concerning about the latter, was the fact that one of the cases did not have any history of travel or evidence of importation. Some of these cases may well differ in provenance to the Indian outbreak cases. Indeed, treatment unresponsive cases of dermatophytosis have been uncommonly associated with *T.rubrum* infections and infections in patients with immunodeficiency, but generally these are rare.^16^, ^17^ Furthermore, most centres across the world do not routinely perform antifungal susceptibility testing (AFST) suggesting many cases are likely to be undetected, especially if clinical is suspicion low. There is a lack of data on species specific breakpoints relating to dermatophyte resistance, further complicating the situation.^18^

To address these challenges, there is a pressing need for robust data collection and studies on species identification from diverse geographic locations and patient groups. Understanding the global spread of suspected and confirmed cases of dermatophyte antifungal resistance is a crucial first step. We have therefore set out to conduct a survey amongst dermatologists worldwide, outside Europe, to map suspected or confirmed cases of recalcitrant and recurrent tinea of glabrous skin and gather more information on the clinical characteristics of such cases.

## METHODS

A standardized questionnaire (see Appendix S1) was distributed to dermatologists and other colleagues internationally through the authors' contact lists between February 2022 and July 2023.

The questionnaire focussed on clinicians' experiences of patients with proven or suspected tinea of the of the *glabrous* skin over the preceding 3 years. Cases were classified into recalcitrant infections (‘patients who have failed to respond to standard first line topical or oral antifungal therapies (including standard dose and duration that would typically clear the infection) or a prolonged period of treatment, that is, more than one month, using a topical antifungal cream or ointment’) or recurrent (‘patients whose infections have relapsed within 4 weeks of completing standard first line topical or oral anti‐fungal therapies (including standard dose and duration that would typically clear the infection completely)’).

Additional questions explored an estimation of the number of such cases seen, most commonly affected body sites, patient's potential exposure to previous treatments, treatment strategies and successes, information on fungal species, laboratory confirmation of resistance and the patient travel history (Appendix S1).

Data were collated and analysed using an online tool (Microsoft Excel). Duplicate or incorrectly completed entries were not included within the analysis. Those respondents who had not seen patients with proven or suspected recalcitrant or recurrent tinea of the *glabrous* skin did not have any subsequent responses analysed. Tinea capitis and tinea unguium cases were excluded because of the large numbers and other reasons for non‐responding to antifungals, such as dermatophytomas and structural nail abnormalities.

## RESULTS

**FIGURE 1:**
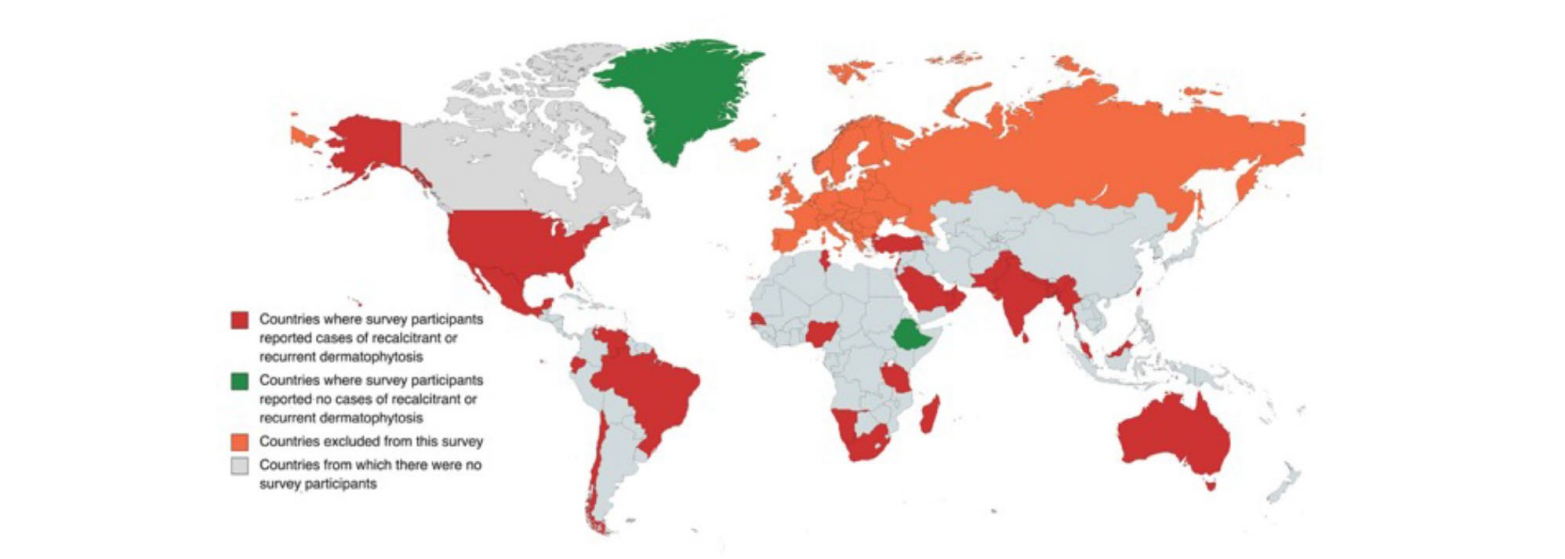
Colour‐coded map representing the countries from where dermatologists who responded to the survey were based.

In total, there were 260 responses from dermatologists working in 36 different countries outside Europe. Responses were from all five of the WHO regions included (Africa, Southeast Asia, the Americas, Eastern Mediterranean and Western Pacific). The sixth WHO region, Europe, was excluded as this was included in a previously published study by two of the three authors of this paper.^14^ Of these, respondents from 94.4% (*n* = 34/36) of surveyed countries outside Europe reported seeing cases of recalcitrant or recurrent dermatophytosis within the preceding 3 years (in red on map, Figure 1). Only two countries had responses that reported seeing no such cases at all (Ethiopia and Greenland in green on map, Figure 1).

Over half of all responses were from dermatologists based in India (55%, *n* = 143), known as Group 1, and remaining responses (45%, *n* = 117) were from dermatologists spread across 35 other countries around the world, outside Europe known as Group 2.

### Recalcitrant cases of tinea

The vast majority of dermatologists surveyed from Group 1 reported seeing cases of tinea corporis or cruris that failed to respond to standard first line therapies for an appropriate duration over the preceding 3 years (93%, *n* = 133/143) and only a minority reported seeing no such cases (6.9%, *n* = 10/143).

The majority of Group 2 reported similar experiences, with 86.3% (*n* = 101/117) stating that they had encountered cases of tinea corporis or cruris that failed to respond to standard first line therapies for an appropriate duration over the preceding 3 years.

When it came to reporting approximately how many such cases were seen over the preceding 3 years, a greater proportion of dermatologists from Group 1 reported seeing ‘more than 20 cases’ (73.7%, *n* = 98/133) than those from Group 2 (42.6%, *n* = 42/101). Although just under half of dermatologists surveyed from Group 2 reported seeing ‘more than 20’ such cases in the preceding 3 years, just over one quarter reported seeing ‘less than 5’ such cases (26.7%, *n* = 27/101).

### The anatomical distribution of the infection

**FIGURE 2:**
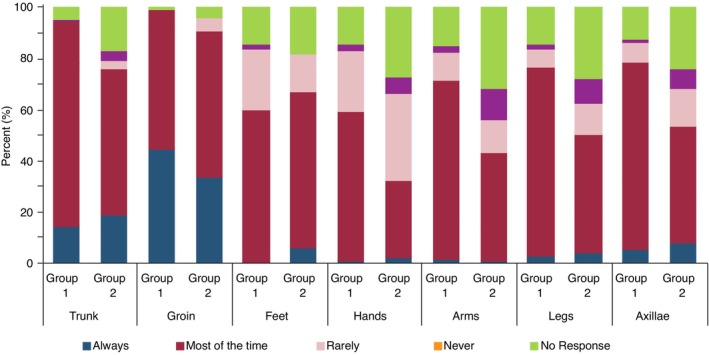
Frequency of anatomical site involvement of recalcitrant cases of dermatophytosis reported by Groups 1 and 2.

**FIGURE 3:**
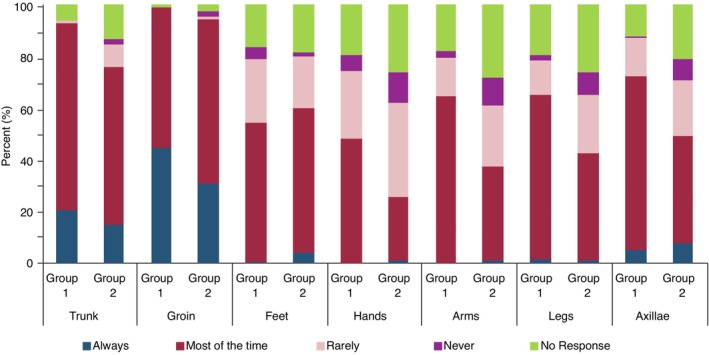
Frequency of anatomical site involvement of recurrent cases of dermatophytosis reported by dermatologists in Groups 1 and 2.

Similar patterns of anatomical disease distribution were reported by both cohorts with the trunk/ tinea corporis and the groin/ tinea cruris being the most commonly reported affected site (see Figures 2 and 3).

### Previous use of topical steroids and antifungals

When asked if their patients had previously used topical steroids or/and antifungals, 84.2% (*n* = 112/133) of Group 1 and 74.3% (*n* = 75/101) of Group 2 stated ‘yes’. Of the 112 dermatologists in Group 1 that answered ‘yes’, 69.6% (*n* = 78/112) stated that they observed previous topical steroid and/or antifungal usage in over half of the patients they had seen with recalcitrant dermatophytosis. Of the 75 dermatologists in Group 2 who stated that they had observed previous topical steroid and antifungal usage in patients with recalcitrant disease, 56% (*n* = 42/75) reported this in over half of patients.

### Recurrent cases of tinea

The vast majority of Group 1 reported seeing cases of tinea of the *glabrous* skin that relapsed after standard first line therapies (95.1%, *n* = 136/143). Similar numbers within Group 2 reported seeing such cases (92.1%, *n* = 93/101).

Over two thirds of Group 1 reported seeing over 20 such cases in the past 3 years, but there was a much more even distribution on reported number of cases seen by Group 2 (<5 cases: 26.9%; *n* = 25/93, 6–10 cases: 24.7% (*n* = 23/93); 11–15 cases: 16.1% (*n* = 15/93); >20 cases: 32.3% (*n* = 30/93)).

### The anatomical distribution of the infection

In a similar pattern distribution to cases of dermatophytosis that failed to respond to therapy, cases that recurred or required longer treatment were reported to involve the trunk and groin most commonly by both cohorts of surveyed dermatologists (See Figures 2 and 3).

### Previous use of topical steroids and antifungals

With regard to previous topical steroids and/or antifungals usage in patients with recalcitrant disease, 81.1 6% (*n* = 111/136) of Group 1 and 68.8% (*n* = 64/93) of Group 2 reported that they had observed this in patients.

Of those that reported previous topical steroid or antifungal usage, 65.8% (*n* = 73/111) of Group 1 and 45.3% (*n* = 29/64) of Group 2 noted this to be the case in over half of their patients with recurrent dermatophytosis.

### Previous oral antifungal

The most commonly reported associated oral antifungals (‘>15 cases’) were itraconazole (34.3%, *n* = 48/140) and terbinafine (28.6%, *n* = 40/140) amongst Group 1. The remaining 52/140 did not provide this information. Similarly, the most commonly reported oral antifungals (‘>15 cases’) associated with suspected or confirmed dermatophyte resistance amongst Group 2 were also itraconazole (15.0%, *n* = 16/107) and terbinafine (10.3%, *n* = 11/107).

Both Groups 1 and 2 reported final treatment success rates of 78.6% (*n* = 110/140) and 66.4% (*n* = 71/107), respectively.

### Dermatophyte species involved and antifungal susceptibility testing

*Trichophyton (T.) mentagrophytes* was by far the most commonly reported species of dermatophyte infection (*n* = 35) reported, followed by *T. rubrum* (*n* = 13) within Group 1. There was one respondent in Group 1 who reported *T. indotineae*. Interestingly, *T. rubrum* was the most frequently noted implicated species within Group 2 (*n* = 14), followed by *T. mentagrophytes* (*n* = 5). There were some responses that just noted ‘*Trichophyton* species’ (*n* = 3). Without molecular tools, *T.indotineae* is likely to be identified on morphological grounds as *T. mentagrophytes* or *T. interdigitale*.

Dermatophyte resistance was noted to have been confirmed by 7.1% (*n* = 10/140) and 8.9% (*n* = 9/107) of dermatologists surveyed in Group 1 and Group 2, respectively. Although 10 dermatologists based in India (Group 1) stated that they were able to confirm the presence of dermatophyte resistance, only 5 were able to state by which method. Similarly, 9 dermatologists from Group 2 stated that they were able to confirm dermatophyte resistance but only one stated by which method used.

### Information of the patients' travel histories

A total of 11.8% (*n* = 11/93) of dermatologists from Group 2 noted a history of overseas travel with regard to cases of recalcitrant or recurrent dermatophytosis. Countries or regions that were noted included: India, Pakistan, Bangladesh, Malaysia, United Arabic Emirates and Africa. The vast majority reported that they were ‘unsure’ (57%, *n* = 53/93) if there was any history of overseas travel in such cases.

## DISCUSSION

Rising global rates of dermatophytosis continue to impose a considerable burden on dermatology and primary care facilities on the sub‐continent. Additionally, they are becoming increasingly more difficult to treat and clinically more extensive. There are multiple factors implicated in the rising number of recalcitrant and recurrent dermatophyte infections, but arguably the most concerning, is the rise in the emerging newly identified *T. indontineae* and its association with confirmed antifungal resistance against terbinfaine.^6^, ^7^, ^8^

The earliest report of dermatophyte resistance first emerged in the 1960s, and for some time after this, it was considered a rare entity.^19^ However, recent years have seen a sharp rise in the number of such cases being reported from centres on the sub‐continent and plethora of such cases published within the international literature.^13^, ^14^, ^15^ Yet, the insufficient availability of diagnostic facilities across various regions around the world hinders our ability to establish the full scope of this resistance and our understanding of this entity.

We therefore set out to conduct this survey of international dermatologists to improve our understanding of the extent of recalcitrant and recurrent tinea of the *glabrous* skin, its clinical features and associated characteristics.

We obtained 260 survey responses from dermatologists across 36 countries outside Europe.

A higher proportion of respondents from India (Group 1) reported observing an increased number of cases (>20 over the past 3 years) than those in Group 2.

Another interesting observation is the breakdown of locations where individuals reported seeing ‘more than 20 cases’ of either recalcitrant or recurrent dermatophytosis in the past 3 years. These countries were Bahrain (1/1), Bangladesh (19/21), Israel (1/2), Maldives (1/1), Mexico (1/3), Nepal (4/6), Saudi Arabia (1/1), Qatar (1/1), Pakistan (8/19), UAE (3/5), Sri Lanka (1/1) and Lebanon (1/11). With the exception of Mexico, the geographical adjacency of these nations to India, the probable epicentre of this ‘outbreak’, is noteworthy.

Respondents from Group 1 and Group 2 described comparable clinical features, most notably the most common anatomical sites being involved as the trunk and groin, a finding consistent with the literature from authors observing the outbreak in India.^6^

Both Groups 1 and 2 reported a large portion of patients with tinea cruris, which may include tinea genitalis (tinea of pubic hair and genitals), another rising entity on the sub‐continent. An important element to consider here is the possibility of sexually transmitted *T. indontineae*, in the form of tinea cruris or tinea genitalis, similar *to T. mentagrophytes* type VII, as proposed by Luchinger et al.^20^ This may be accelerating the spread. Additionally, the lack of regulation of over‐the‐counter antifungal and potent steroid combination therapies and their misuse has also been implicated in the rise of cases of tinea genitalis in India.^21^

Within this study, there were high rates of reported previous topical steroid and antifungal combination therapy usage by patients with recalcitrant dermatophytosis in Group 1. Notably, there were lower rates of previous topical steroid and antifungal therapy usage in cases of recurrent dermatophytosis for Groups 1 and 2. Our findings also underscore issue of widespread availability of over‐the‐counter topical steroid and antifungal combinations that warrant regulation.

Another alarming finding from this survey is the high rate of previous oral itraconazole usage that has been reported. Of note, there was a higher rate of previous oral itraconazole use than oral terbinafine use reported by Group 1. Antifungal resistance to terbinafine has been well documented within the literature but resistance to itraconazole less so.^22^ One explanation might be that the local Indian guidelines recommend itraconazole as one of the first line treatments in patients with recalcitrant and recurrent dermatophyte infections.^23^ This highlights the need for further research and development for alternate therapeutic strategies.

It is interesting to note that *T. mentagrophytes* (*n* = 35) was more commonly reported as the implicated species followed by *T. rubrum* (*n* = 13) by Group 1. This is also reflected in the literature, which notes that the predominant dermatophyte species in India is now *T. mentagrophytes*.^24^ Given the rise in number of difficult to treat cases of dermatophytes and this shift in predominant species, one must question whether or not a significant portion may in fact be *T. indotineae*. This is the species most commonly associated with terbinafine resistance and is indistinguishable from *T. mentagrophytes* on culture and can only be identified using molecular methods,^18^, ^24^ although with further study it will be important to separate the *T. indotineae* cases from the previously rare cases of recalcitrant tinea corporis/cruris due to *T. rubrum*.

A high proportion of dermatologists outside India, in Group 2, were noted to be ‘unsure’ of a travel history. This suggests potential gaps in awareness of this entity and its associated risk factors or recall bias. Nonetheless, this highlights the need for improved dermatologist training and understanding of this disease.

Our study includes a large number of survey responses, with a balanced representation from both India (*n* = 143) and non‐European countries (*n* = 117) that span all five of the WHO regions included in this study (Africa, Southeast Asia, the Americas, Eastern Mediterranean and Western Pacific). Nonetheless, it is not without its limitations. Recall bias, language barriers (with the survey only in English) and the exclusion of onychomycosis cases (which would have constituted a significant proportion) limit our findings. It is imperative to mention the absence of or limited survey responses from significantly large regions like China, South America and Africa, which leaves gaps in our knowledge. Incorporating such large geographical regions could have presented a more holistic understanding, even if no such cases are being seen in these regions. Despite no responses from China for instance within this survey, authors have since published the first confirmed case of terbinafine resistant *T. indontineae*.^25^

In summary, this study adds to the growing body of evidence that demonstrates the far reaching spread of clinically suspected or confirmed antifungal resistance of dermatophytes. We have been able to find comparisons between what is being reported by dermatologists based in India and those based internationally, outside of Europe. A pressing need now arises from our study for a standardized method of prospectively collecting data on recalcitrant, recurrent or proven resistant dermatophytosis cases, emphasizing the requirement for a global disease registry. Enhanced diagnostic tools, antifungal susceptibility testing (AFST) and establishment of clinical breakpoints are also paramount.

In conclusion, our study underscores the need for global collaboration, awareness and enhanced diagnostic capabilities globally to better understand and manage the threat of emerging dermatophyte resistance.

## FUNDING INFORMATION

No funding was obtained for this work.

## CONFLICT OF INTEREST STATEMENT

SSK has been awarded a grant from the Women's Dermatological Society and GLODERM, which contributes to part of her salary and has received speaker fees/travel support from LEO Pharma, Janssen, AbbVie and L'Oréal. DML has received honoraria as a consultant for advisory board meetings by AbbVie, Janssen, Sanofi, LeoPharma, Novartis and as a speaker and/or received grants from the following companies: Abbvie, Janssen, Novartis, Sanofi, Jamjoom Pharma, UCB and Leo Pharma during the last 3 years. RH has no conflicts of interest to declare.

## ETHICAL APPROVAL

Not applicable.

## ETHICS STATEMENT

Not applicable.

## Supporting information


Appendix S1.

## Data Availability

The data that support the findings of this study are available from the corresponding author upon reasonable request.
