# Supplementary material for: An international survey of recalcitrant and recurrent tinea of the glabrous skin—A potential indicator of antifungal resistance
Source: J Eur Acad Dermatol Venereol. 2024 Jul 13;39(6):1185–91. doi: 10.1111/jdv.20146 (PMC12105417; doi:10.1111/jdv.20146)
Supplement: Supplementary file 1 — Appendix S1. [file JDV-39-1185-s001.pdf]

Anti-fungal treatment resistance +/- failure questionnaire

* Indicates required question

1. Please enter your name *

____

2. Please enter your work place/ base institution *

____

3. Please enter the country you are currently based in *

____

4. 1. In the past 3 years have you seen patients with proven or suspected tinea corporis or cruris whose infections have failed to respond to standard first line topical or oral antifungal therapies (including standard dose and duration that would typically clear the infection) or a prolonged period of treatment, ie more than one month, using a topical antifungal cream or ointment? *

Mark only one oval.

- ( ) Yes
- ( ) No

5. 1 (a) If you have answered "Yes" to Q1, please state approximately how many such cases you have seen in the past 3 years?

Mark only one oval.

- <5 cases
- 6-10 cases
- 11-15 cases
- >20 cases

6. 1. (b) If you have answered “Yes” to Q1, please indicate how frequently each body site was involved?

Tick all that apply.

	Always	Most of the time	Sometimes	Rarely	Never
**Trunk**	☐	☐	☐	☐	☐
**Groin**	☐	☐	☐	☐	☐
**Feet**	☐	☐	☐	☐	☐
**Hands**	☐	☐	☐	☐	☐
**Arms**	☐	☐	☐	☐	☐
**Legs**	☐	☐	☐	☐	☐
**Axillae**	☐	☐	☐	☐	☐

7. 1. (c) If you have answered, "Yes" to Q1, had they been using a topical anti-fungal/ steroid combination cream

Mark only one oval.

- Yes
- No
- Unsure

8. 1. (c) (i) If you answered "Yes" to Q1 (c), please indicate what proportion of patients were suspected to have been exposed to a topical anti-fungal/ steroid combination?

Mark only one oval.

- < 25% had been exposed
- 25-50% had been exposed
- >50% had been exposed

9. 2. In the past three years have you seen patients with suspected or proven tinea corporis or cruris whose infections have relapsed within 4 weeks of completing standard first line topical or oral anti-fungal therapies (including standard dose and duration that would typically clear the infection completely) ? *

Mark only one oval.

- Yes
- No

10. 2 (a) If you have answered "Yes" to Q2, please state approximately how many such cases you have seen in the past 3 years?

Mark only one oval.

- <5 cases
- 6-10 cases
- 11-15 cases
- >20 cases

11. 2. (b) If you have answered “Yes” to Q2, please indicate how frequently each body site was involved?

Tick all that apply.

	Always	Most of the time	Some of the time	Rarely	Never
**Trunk**	☐	☐	☐	☐	☐
**Groin**	☐	☐	☐	☐	☐
**Feet**	☐	☐	☐	☐	☐
**Hands**	☐	☐	☐	☐	☐
**Arms**	☐	☐	☐	☐	☐
**Legs**	☐	☐	☐	☐	☐
**Axillae**	☐	☐	☐	☐	☐

12. 2. (c) If you have answered, "Yes" to Q2, had they been using a topical anti-fungal/ steroid combination cream *

Mark only one oval.

- Yes
- No
- Unsure

13. 2. (c)(i) If answered “Yes” to Q2(b) please what proportion of patients were suspected to have been exposed to a topical anti-fungal/ steroid combination?

Mark only one oval.

- <25% had been exposed
- 25-50% had been exposed
- >50% had been exposed

If you have answered “Yes” to question 1 and/ or 2, please answer questions 3- 7. If you answered "No" to both question 1 and 2, please do not answer questions 3-7 and submit your questionnaire.

14. 3. Which of the following first line oral anti-fungal agent(s) was (were) used and on how many occasions in the past 3 years, to the best of your memory, was it associated with suspected or confirmed anti-fungal resistance +/- treatment failure

Tick all that apply.

	<5 cases	6-10 cases	11-15 cases	>15 cases	No cases
**Terbinafine**	☐	☐	☐	☐	☐
**Itraconazole**	☐	☐	☐	☐	☐
**Griseofulvin**	☐	☐	☐	☐	☐
**Ketaconazole**	☐	☐	☐	☐	☐

15. 4. Was (were) your patient(s) with suspected or confirmed anti-fungal resistance and/ or anti-fungal treatment failure finally treated successfully?

Mark only one oval.

- ◯ Yes
- ◯ No

16. (a) If you answered “Yes, what treatment was used?

____

17. 5. What dermatophyte species, if known, have been involved in cases of suspected or confirmed anti-fungal resistance and/or treatment failure?

18. 6. Was dermatophyte resistance in such cases ever confirmed by laboratory test?

Mark only one oval.

- Yes
- No

19. (a) If you answered “Yes”, what test was used to confirm this?

20. (b) What anti-fungal testing methods are available in your country?

21. 7. Was there any history of travel in such cases?

Mark only one oval.

- Yes
- No
- Unsure

22. (a) If “Yes”, what country/ countries did the patient visit?

This content is neither created nor endorsed by Google.

Google Forms
